# Novel Oxidative Ring Opening Reaction of 1*H*-Isotelluro-chromenes to Bis(*o*-formylstyryl) Ditellurides

**DOI:** 10.3390/molecules15031466

**Published:** 2010-03-09

**Authors:** Haruki Sashida, Hirohito Satoh, Kazuo Ohyanagi, Mamoru Kaname

**Affiliations:** 1 Faculty of Pharmaceutical Sciences, Hokuriku University, Kanagawa-machi, Kanazawa 920-1181, Japan; E-Mail: m-kaname@hokuriku-u.ac.jp (M.K.); 2 Faculty of Pharmacy, Institute of Medical, Pharmaceutical and Health Sciences, Kanazawa University, Kakuma-machi, Kanazawa 920-1192, Japan; E-Mail: ohyanagi@p.kanazawa-u.ac.jp (K.O.)

**Keywords:** isotellurochromene, *m*CPBA oxidation, 2-benzotelluropyrylium salt, distyryl ditelluride

## Abstract

The oxidation of 1-unsubstituted or 1-phenyl-1*H*-isotellurochromenes with *m*‑chloroperbenzoic acid (*m*CPBA) in CHCl_3_ resulted in a ring opening reaction to produce as the sole products the corresponding *o-*formyl or benzoyl distyryl ditellurides, which were also produced by the hydrolysis of the 2-benzotelluropyrylium salts readily prepared from the parent isotellurochromene.

## 1. Introduction

The preparation and investigation of the reactions of new tellurium-containing heterocycles have lately attracted increasing interest in the fields of both heterocyclic [1,2,3] and organotellurium chemistry [4,5,6,7,8]. We have been studying the syntheses and reactions of the novel tellurium- or selenium-containing heterocyclic compounds over the past twenty years. Our synthetic strategy [9,10] for the preparation of these compounds is based on the intramolecular cyclization of the tellurol or selenol moieties to a triple bond. As a part of our continuing studies, we have previously reported the successful synthesis of the isotellurochromenes as the precursors for the preparation of the novel 2-benzotelluropyrylium salts [11,12]. In addition, the 1*H*-isotellurochromenes were found to transform into the 1,2-dihydro-2-metalanaphthalenes *via* (*E*)-*o*-(2'-lithiovinyl)benzyllithium generated by a tellurium-lithium exchange reaction [13]. In this study, an investigation of the oxidation of the isotellurochromenes is described as an extension of the known reactivity of these compounds.

There are several reports concerning the oxidation of mono and benzene ring fused six-membered heterocycles containing a chalcogen atom. The selenium dioxide oxidation of 4*H*-thiopyrans [14] and 4*H*-selenopyrans [14] produces a ring contraction to give hiophenes and selenophenes, respectively. The oxidation of dihydro-2*H*-selenopyrans [15] with sodium periodate is also known to produce selenophenes. Furthermore, the oxidation of thiochromenes [16], selenochromenes [16] and tellurochromenes [17] by selenium dioxide, trityl perchlorate or K_2_CrO_7_ produced the corresponding 2-formylbenzo[*b*]-thiophenes, -selenophenes and -tellurophenes, respectively. Whereas the isothiochromenes [18,19] are easily oxidized by *m*CPBA to afford the thermally stable normal *S*-oxide products, a similar oxidation of the highly substituted isoselenochromenes [20] produces the corresponding benzo[*b*]selenophene derivatives as the main product. These facts prompted us to investigate the oxidation of the isotellurochromenes **1**.

## 2. Results and Discussion

When *tert*-butylisotellurochromene **1a** was oxidized with 1.2 equiv. of *m*CPBA in CHCl_3_ at 0 °C, the starting material immediately disappeared, and the bis(*o*-formylstyryl) ditelluride **2a** was obtained in 83% yield as the sole product after the usual work up. The structure of the ditelluride **2a** was characterized by NMR, IR and mass spectroscopy. The low-mass spectrum of this compound suggested the ditelluride molecular formula based on the molecular ion at *m/z* = 634 (^130^Te) and the expected isotope pattern for Te_2_. In addition, the HRMS of **2a** showed the same exact molecular formula. Similarly, the 1-nonsubstituted **2b**, alkyl **2c-g** and phenyl **2h** derivatives were also obtained in moderate to good yields. These results are summarized in Table 1 (entries 1-8). The double bond in the products **2** was proven to have a (*Z*)-stereochemistry by the vicinal coupling constant (*J* = 10 Hz) in the ^1^H-NMR spectra of the nonsubstituted derivative **2b**. A similar *m*CPBA oxidation of the 1-substituted isotellurochromenes **1** was examined (entries 9, 10). The 1-phenylisotellurochromene **1i** [21] was oxidized to give the bis(*o*-benzoylstyryl) ditelluride **2i **in 80% yield, while the similar oxidation of the 1-methyl isochromene **1j** [21] resulted in decomposition to afford a complex mixture without any characterized products. The significant differences in the product yield according to whether or not the 1-substituent is a methyl group cannot be clearly explained at the present time. The structure of the products **2** including the geometry of the styryl moiety was finally determined by an X-ray crystallographic analysis using the *tert*-butyl derivative **2a**. 

**Table 1:** Oxidation of isotellurochromenes **1** with *m*CPBA. 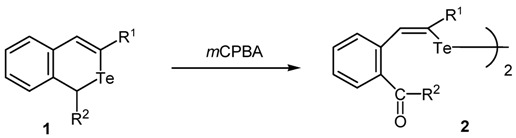

Entry	Product	R^1^	R^2^	Yield (%)	Appearance (mp, °C)
1	**2a**	*t*-Bu	H	83	orange prisms, 140–143
2	**2b**	H	H	43	orange prisms, 95–97
3	**2c**	Me	H	57	orange oil
4	**2d**	*n*-Pro	H	60	orange oil
5	**2e**	*n*-Bu	H	70	orange oil
6	**2f**	1-cyclohexenyl	H	83	orange oil
7	**2g**	*n*-Oct	H	62	orange oil
8	**2h**	Ph	H	67	orange oil
9	**2i**	*t*-Bu	Ph	80	orange oil
10	**2j**	*t*-Bu	Me	0	---

The telluroxides **3**, which are presumed to be produced by the oxidation of the isotellurochromene **1** with *m*CPBA in this reaction, were too unstable to isolate. The oxidation of **1a** with *m*CPBA, followed by treatment with Ac_2_O resulted in a Pummerer-type rearrangement to afford 1-acetoxy-isotellurochromene **4a** as a pale yellow oil, together with a small amount of the ditelluride **2a**. The hydrolysis of the acetoxy derivative **4a** with NaHCO_3_ aq. produced the ditelluride **2a** in almost quantitative yield.

In addition, the ditellurides **2a** and **2b** could be obtained in high yields by the hydrolysis of the 2-benzotelluropyrylium tetrafluoroborates **5** [15], which had previously been prepared by the treatment of the isotellurochromenes **1** with triphenylcarbenium tetrafluoroborate (Ph_3_C^+^ BF_4_^-^) in MeNO_2_ in almost quantitative yields. The initial intermediates **6** were produced by the nucleophilic attack of water at the C-1 position of the telluropyrylium cation **5**, and then oxidized to give **2**. The ditelluride **2a** was easily converted to the tellulium trichloride **8** by fission of the Te-Te bond by sulfuryl chloride in benzene in quantitative yield.

**Figure 1:**
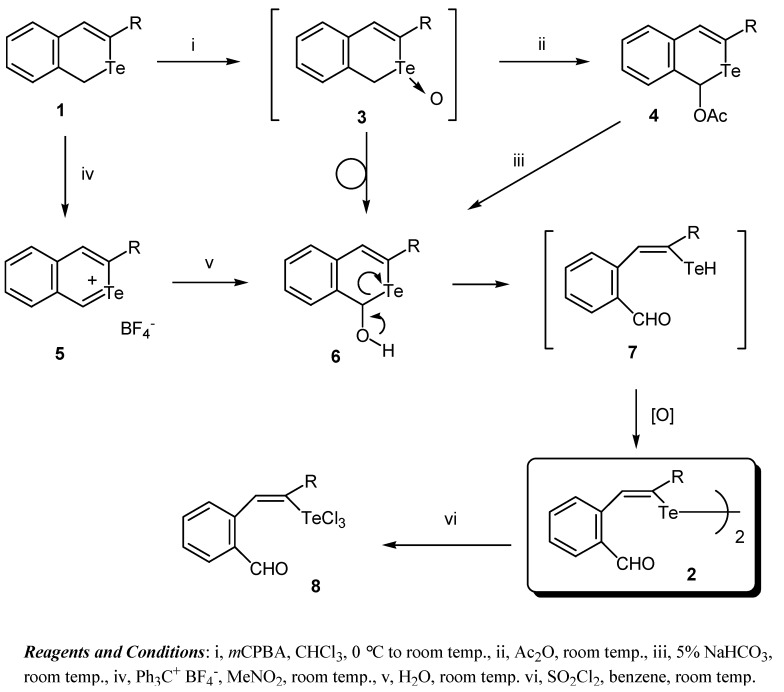
Reaction conditions and a plausible mechanism.

A plausible mechanism for the formation of the ditellurides **2** from the isotellurochromenes **1** and 2-benzotelluropyrylium salts **5** is outlined in Figure 1. The telluroxides **3** generated by the oxidation of the isotellurochromenes **1** rearranged to afford the 1-hydroxyisochromenes **6** by a Pummerer-type reaction. The intermediate **6** undergoes ring opening with migration of the hydroxy proton to form the vinyltellurol **7**. The resulting tellurol tautomer** 7** is immediately oxidized by *m*CPBA or air to give the distyryl ditelluride **2**.

In summary, the oxidation of the isotellurochromenes with *m*CPBA resulted in the unprecedented ring-opening reaction to give the bis[*o*-formyl(benzoyl)-α-styryl] ditellurides in moderate to good yields. The reaction might proceed through a Pummerer-type reaction *via* the telluroxides. Further detailed studies of this reaction, an expanding the applications and selenium analogues are now in progress.

## 3. Experimental

Melting points were measured on a Yanagimoto micro melting point hot stage apparatus and are uncorrected. IR spectra were determined with a Horiba FT-720 spectrometer. Mass spectra (MS) and HRMS were recorded on a JEOL SX-102A instrument. NMR spectra were determined with a JEOL ECP- 500 (500 MHz) spectrometer in CDCl_3_ using tetramethylsilane as internal standard and *J* values are given in Hz. Microanalyses (MT-5) were performed in the Microanalytical Laboratory in this Faculty. The starting materials, isotellurochromenes (**1**) were prepared by the literature method [12].

*Oxidation of 3-tert-butylisotellurochromene* (**1a**) *with mCPBA: formation of (Z)-bis(o-formyl-α-tert-butylstyryl) ditelluride* (**2a**)*: m*CPBA (70%, 296 mg, 1.2 mmol) was added to a solution of 3-*tert*-butylisotellurochromene (**1a**, 302 mg, 1.0 mmol) in dry CHCl_3_ (10 mL) at 0 °C and the mixture was stirred at room temperature for 1h. After addition of sat. NaHCO_3_ aq. (10 mL), aqueous mixture was extracted with CH_2_Cl_2_ (50 mL x 3). The organic layers were washed with brine (30 mL × 2), dried (MgSO_4_) and evaporated. The resulting residue was chromatographed on silica gel using *n*-hexane-CH_2_Cl_2_ (2:1) as an eluent to give pure **2a** (263 mg, 83%), dark red prisms, mp 140-143 °C (from acetone-hexane); MS *m/z* (%) : 634, 632, 630, 628 (1, 2, 2, 1, M^+^), 187 (100), 145 (33), 115 (22). IR (KBr) cm^-1^ 1690 (C=O); ^1^H-NMR (500 MHz, CDCl_3_) δ 1.24 (18H, s, *t*-Bu × 2), 6.88 (2H, s, Ph-C*H*=C- × 2), [7.20 (2H, d, *J* = 7.6 Hz), 7.36 (2H, dd, *J* = 7.7, 7.7 Hz), 7.47 (2H, ddd, *J* = 7.7, 7.6, 1.4 Hz), 7.80 (2H, dd, *J* = 7.7, 1.4 Hz) Ph-H], 10.06 (2H, s, CHO × 2). ^13^C-NMR (125 MHz, CDCl_3_) δ 30.7 (q), 41.3 (s) 127.5 (d), 128.3 (d), 131.6 (d), 133.5 (s), 133.6 (d), 134.6 (s), 138.0 (s), 144.4 (s), 191.5 (s); HRMS *m/z* Calcd for C_26_H_30_^130^Te_2_: 634.0374. Found: 634.0352.

## Appendix

### Appendix 1

This paper constitutes Part 28 in the ‘Studies on Tellurium-Containing Heterocycles’, For Part 27: Sashida, H.; Nakabayashi, S.; Kaname, M., Minoura, M. 2-Substituted Isotellurochromenium Salt Derivatives: Preparations, Structures, Spectroscopic Properties. *Heterocycles* **2010**, *80*, 1339–1352.

### Appendix 2

The details of the X-ray data of bis(*o*-formylstyryl) ditelluride **2a** will be reported in a full paper that will appear in the near future.
